# A Laminar Microfluidic Platform for Probing the Effects of Spatially Heterogeneous Drug Distributions

**DOI:** 10.3390/mi17060655

**Published:** 2026-05-26

**Authors:** Yang Zeng, Wenyan Liu, Jiahao Fu, Bingchen Che, Yonggang Liu, Xiaobo Gong, Dan Sun, Ce Zhang

**Affiliations:** 1 State Key Laboratory of Photon-Technology in Western China Energy, Shaanxi Provincial Key Laboratory of Optoelectronic Technology, Institute of Photonics and Photon-Technology, Northwest University, No. 1, Xuefu Avenue, Xi’an 710127, China; 15508376930@163.com (Y.Z.); 16673836751@163.com (W.L.); 15565465083@163.com (J.F.); 0325abcde@163.com (B.C.); 2 School of Physics, Northwest University, No. 1, Xuefu Avenue, Xi’an 710127, China; 3 Huaxin Micro-Fish (Suzhou) Biotechnology Co., Ltd., No. 99-4-301-01, Fuda Road, Taicang 215411, China; 4 Beijing National Laboratory for Condensed Matter Physics, Institute of Physics, Chinese Academy of Sciences, Beijing 100190, China; 5 Laboratory of Stem Cell and Tissue Engineering, Chongqing Medical University, Chongqing 400016, China; 6 School of Ocean and Civil Engineering, Shanghai Jiao Tong University, Shanghai 200240, China

**Keywords:** microfluidic chip, spatial heterogeneity, laminar-flow microfluidics

## Abstract

We herein designed a 64-chamber laminar-flow microfluidic chip with independently addressable culture units capable of establishing spatially heterogeneous chemical environments to mimic tissue microenvironments. Stable chemical gradients were successfully generated within the chip through controlled laminar flow, allowing precise spatial modulation of cellular exposure. Using TNF-α as a model stimulus, we observed a clear time delay in NF-κB activation between cells directly exposed to the cytokine and those located on the medium-only side, confirming the establishment of spatially distinct chemical conditions. Notably, even cells not directly exposed to TNF-α eventually responded, indicating that molecular diffusion along the static solid–liquid interface serves as an effective delivery route for bioactive molecules. To further demonstrate the platform’s utility, we constructed a skin-mimetic co-culture model of HaCaT keratinocytes and human skin fibroblasts (HSFs) to assess the diffusion and cytotoxic effects of 5-fluorouracil (5-FU). The results revealed that fibroblasts provided protective effects against 5-FU-induced cytotoxicity, likely via paracrine signaling or direct cell–cell interactions. These findings highlight the platform’s capacity for probing not only spatial drug-delivery dynamics but also intercellular interactions under physiologically relevant conditions. This system offers a powerful and versatile tool for studying spatiotemporal signaling, drug screening, and topical therapeutic development.

## 1. Introduction

Tissue microenvironments are inherently heterogeneous, with spatial gradients of oxygen, nutrients, and signaling molecules influencing cell behavior [1,2,3,4]. In skin, for instance, the epidermis is more hypoxic than the vascularized dermis due to limited oxygen diffusion from the atmosphere [5]. Similarly, solid tumors exhibit sharp spatial heterogeneity due to abnormal vasculature, leading to distinct niches such as well-perfused rims versus hypoxic, acidic cores [6,7,8]. These gradients profoundly affect cell proliferation, metabolism, and treatment response [9,10].

Such spatial heterogeneity presents a major challenge for drug delivery. Uneven distribution of therapeutics within tissues can create “pseudoresistant” zones, where drug concentration is insufficient to elicit a therapeutic effect despite cellular sensitivity [11,12]. This results in heterogeneous treatment responses, especially in solid tumors, where poorly perfused areas often survive therapy and drive relapse. Even in non-tumor tissues like skin, structural barriers can impede uniform drug penetration, underscoring the need to assess how microenvironmental gradients affect therapeutic efficiency [13,14].

Quantifying these gradients requires validated measurement approaches. In microfluidic systems, flow dynamics and transport parameters are typically characterized by computational fluid dynamics (CFD) simulations, particle image velocimetry (PIV) and fluorescence recovery after photobleaching (FRAP) to confirm laminar flow stability and diffusion-dominated transport [15,16,17,18]. In vivo, tumor and tissue heterogeneity is assessed through intravital oxygen and pH imaging, as well as clinical drug distribution mapping, providing quantitative benchmarks that inform the design of physiologically relevant in vitro models [19,20,21].

To address this, microfluidic systems have emerged as powerful tools for reproducing spatially controlled chemical environments. Under laminar-flow conditions, two or more liquid streams can form stable chemical interfaces with minimal mixing, allowing precise delivery of gradients or localized stimuli [22,23,24]. Unlike traditional static cultures, microfluidics can mimic dynamic tissue conditions, enabling real-time tracking of spatially varying drug exposure and cellular responses [25]. Furthermore, laminar-flow platforms allow for micron-level spatial control over drug exposure. By adjusting flow streams, researchers can selectively treat specific regions of cell cultures, enabling a detailed study of how cells in different spatial positions respond to external stimuli. This strategy has been used to model wound healing, tumor heterogeneity, and drug penetration in engineered tissues [26,27,28].

In this study, we leverage a laminar-flow-based microfluidic chip to systematically investigate how spatial gradients in drug concentration influence heterogeneous cellular responses in a skin-mimetic co-culture model. By seeding human epidermal keratinocytes (HaCaT) and human skin fibroblasts (HSFs) as a monolayer, we replicate the layered architecture of skin and apply 5-fluorouracil (5-FU) through one side of the chamber via stable laminar flow, generating a well-defined chemical interface. This setup allows us to observe how drug molecules diffuse along the substrate and elicit spatially distinct and heterogeneous responses, including differential cell survival and signaling activation. Importantly, we examine how the presence of fibroblasts modulates keratinocyte susceptibility to the drug, simulating protective intercellular interactions seen in vivo. Together, our platform provides a controlled and physiologically relevant system to quantify drug-delivery efficiency and heterogeneous therapeutic outcomes under microenvironmental gradients, offering insights for both topical drug development and cancer therapy.

## 2. Materials and Methods

### 2.1. Design and Fabrication of Microfluidic Device

**Figure 1:**
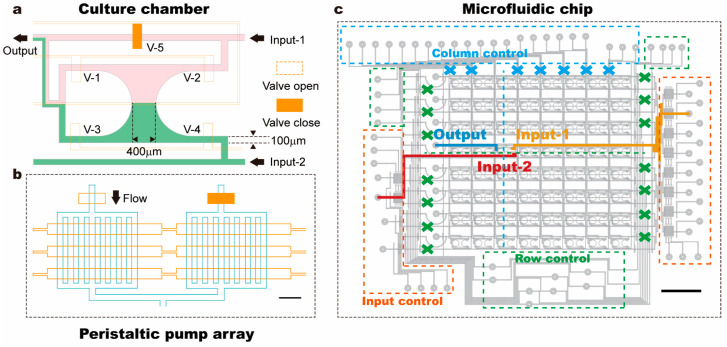
Design of the laminar-flow cell culture chip. (**a**) Schematic diagram of a single chamber shows that the laminar flow can be generated via simultaneous opening of two different inlets, i.e., input-1 and -2. (**b**) As reported previously, each peristaltic pump is composed of 3 valves, the sequential operation of which determines the flow rate (scale bar: 300 μm). (**c**) The microfluidic device contains multiple identical culture chambers, and laminar flow can be formed in each chamber (scale bar: 6 mm).

We designed and fabricated microfluidic devices following the standard PDMS-based soft lithography protocol published elsewhere [29] for basic device fabrication. The chip architecture, together with the specific biological applications described herein, is original to this work. Briefly, we designed a microfluidic device (shown in Figure 1), which contains 64 culture units for the maintenance and simulation of cells in a variable environment, using AutoCAD 2018 (Autodesk Inc., San Rafael, CA, USA) (Autodesk Inc., San Rafael, CA, USA). The device operates on the principle of laminar flow in microchannels, where low-Reynolds-number (Re < 1) conditions prevent turbulent mixing, allowing two parallel fluid streams to form a stable interface within each culture chamber. This interface enables spatially heterogeneous chemical environments by introducing different solutions (e.g., media with or without stimuli) through separate inlets, with diffusion occurring primarily at the boundary due to minimal convective mixing.

The chip consists of a flow layer for fluid transport and a control layer for pneumatic valve actuation, enabling the precise regulation of fluid paths. Each culture chamber measures 25 µm in height and 400 µm in width, with inlet channels 100 µm wide and 25 µm high to ensure uniform flow distribution and minimize shear stress on cells. The overall device footprint is approximately 5 cm × 4 cm, accommodating the 64 chambers arranged in an 8 × 8 array for parallel processing.

The control and flow layer templates are fabricated using standard UV-lithography, using two different photoresists: SU8 3025 (Microchem, Westborough, MA, USA) for the flow layer and AZ50X (AZ Electronic Materials, Luxembourg) for the control layer. SU8 3025 was selected for the flow layer due to its low viscosity and suitability for creating thick, high-aspect-ratio structures (up to 25 µm height) with vertical sidewalls, which are essential for defining precise channel geometries and ensuring laminar-flow stability. In contrast, AZ50X, a positive photoresist, was chosen for the control layer to enable finer patterning resolution required for the pneumatic valves, which demand high precision to achieve reliable sealing without leakage.

For the chip, 55 g of PDMS (10:1 of monomer: catalyst ratio) was mixed, degassed and poured over the trimethylchlorosilane-treated patterned silicon wafer. After 1–2 h of degassing in the vacuum chamber at −0.085 MPa, the PDMS was transferred to the ventilating oven and cured for 60 min at 80 °C. The flow and control layers were then treated by the plasma etching machine, aligned and bonded together using a customized optical device. After 2 h of thermal bonding at 80 °C, the inlet holes were punched using the punching machine. Next, they were bonded to a PDMS-coated coverslip (i.e., the same size as a 96-hole plate) and cured for at least 24 h at 80 °C before use.

### 2.2. Chip Operation

The prepared chip was placed on the stage of the microscope, and the corners were taped to the holder. Control channels were connected to miniature pneumatic solenoid valves (Festo, Lupfig, Switzerland), which were controlled via a custom MATLAB (MathWorks, Natick, MA, USA) program. To optimize the closing pressure of push-up PDMS membrane valves for each chip, the UV-lithography produced a template ranging from 25 to 30 psi.

The system’s operation integrates the control layer (pneumatic lines for valve actuation) with the flow layer (channels for liquid transport). The control layer consists of pneumatic channels filled with pressurized air (25–30 psi), connected to external solenoid valves that regulate air flow to deform PDMS membranes, thereby opening or closing valves in the flow layer. This setup allows selective routing of fluids through the 64 chambers. The flow layer includes inlet ports connected to a peristaltic pump system, which provides continuous liquid delivery. The MATLAB program synchronizes solenoid valve actuation with pump speed, enabling automated sequences for cell loading, medium exchange, and chemical stimulation across multiple chambers.

To support complex experimental designs, input 1 is connected to eight upstream inlets, enabling the simultaneous delivery of multiple chemical solutions into the chip via the peristaltic pump array (Figure 1). The total flow rate from input 1 is determined by the number and combination of active input channels at any given time. To maintain a stable laminar interface within each culture chamber, the peristaltic pump on the input-2 side dynamically adjusts its actuation frequency to match the total flow rate from input 1. This real-time synchronization ensures that the two streams enter the culture chamber at matched velocities, preserving the stability of the laminar-flow interface regardless of the specific chemical composition delivered from input 1.

### 2.3. Cell Culture

HaCaT cells were maintained in Dulbecco’s Modified Eagle Medium (DMEM, D5796, Sigma) supplemented with 10% fetal bovine serum (FBS, F2442, Sigma) and 1% penicillin/streptomycin (P/S). HSF cells were cultured in DMEM/F12 medium containing 10% FBS, 1% P/S, 0.005 mg/mL insulin, 5 ng/mL basic fibroblast growth factor (bFGF), 1 µg/mL hydrocortisone, 50 µg/mL ascorbic acid, and 7.5 mM L-glutamine. Cells were incubated at 37 °C in a humidified atmosphere with 5% CO_2_. NIH 3T3 p65^−/−^ cells were transfected with p65-dsRed and H2B-GFP for tracking and analysis of NF-κB activation and nuclear shape changes. The cells were maintained in DMEM with 10% (*v*/*v*) FBS, 10 U/mL penicillin and 10 μg/mL streptomycin (CAS# 15140-122, Gibco) at 37 °C, >98% humidity, and 5% CO_2_. NIH 3T3 p65^−/−^ cells were used for NF-κB activation assays, whereas HSFs were used for co-culture experiments with HaCaT cells.

### 2.4. Cell Loading and Peristaltic Pump Control

To confirm the function of the chip, two different fluorescent dyes (i.e., fluorescein 5-isothiocyanate (FITC, CAS# 3326-32-7) and Rhodamine B (RhB, CAS# 81-88-9)) were diluted by the deionized water and input from the upper and lower inlet ports. For the NIH 3T3 p65^−/−^ cells, the culture chamber was treated with fibronectin (0.25 mg/mL; Millipore, Austria) for at least one hour, then flushed using either PBS or cell culture media. Cell loading was performed using a semi-automated process controlled by the MATLAB program, which sequentially actuates the solenoid valves to direct cell suspension into the chambers. Cells were resuspended at a density of 10^6^ per milliliter and introduced via the inlet ports using the peristaltic pump to ensure uniform distribution across the chamber floor. The program automates valve opening and closing to fill each chamber individually or in batches, allowing cells to settle and adhere for 4 h before experiments. This approach facilitates a consistent seeding density across the 64 chambers, enabling parallel replicates for high-throughput testing while focusing data analysis on representative chambers. The experimental design leverages this integrated system to achieve complex, automated workflows. For instance, the valve–pump combination enables precise implementation of laminar flow setups by routing dyes or stimuli to specific inlets while maintaining isolated chambers. These are realized through programmed sequences, e.g., initial cell loading via batch valve opening, followed by medium perfusion and then selective introduction of chemicals via individual inlet control, all monitored in real-time via microscopy.

The peristaltic pump timing sequence was adopted as the fluid-driving parameter for all stimulation experiments. Each control state lasted 1/12 s (valve-switching frequency: 12 Hz); as one complete pump cycle comprises six states, the full pump-cycle frequency was 2 Hz. The chamber-adjacent valves served only for target-chamber selection and local flow-path switching, not for direct flow-rate control.

### 2.5. TNF-α Stimulation and NF-κB Activation Assay

To elucidate the spatiotemporal dynamics of TNF-α-induced NF-κB activation within a spatially heterogeneous microenvironment, we employed a microfluidic platform that allows precise control over localized chemical stimulation. NIH 3T3 p65^−/−^ cells, which were transfected with p65-dsRed and H2B-GFP to enable real-time monitoring of NF-κB activation and cell growth, were cultured under standard conditions. Exponential-growth cells were used throughout chip experiments. The cells were seeded in a culture flask at a density of 5 × 10^5^ cells mL^−1^ and cultured for 24 h. Cells were harvested at 80% confluence with trypsin, resuspended and loaded into chips through a semi-automated loading program at a cell density of 10^6^ per milliliter, depending on the desired cell density. After allowing cells to adhere and adjust on the culture chambers for 4 h, tumor necrosis factor-alpha (TNF-α) was then flown into the chamber for stimulation. The environmental conditions were maintained using a temperature control and incubator system (Huaxin Micro-fish, China) at strictly 37 °C, >98% humidity and 5% CO_2_ during the experiment, and the PDMS chip was covered with a stage-top incubator connected to a humidifier and a gas exchanger.

### 2.6. Live-Cell Fluorescence Microscopy

For image acquisition, a Nikon Ti2-E inverted microscope with an automated translational stage and a digital complementary metal-oxide semiconductor (CMOS) camera (ORCAFlash4.0, Hamamatsu, Japan) and our customized live-cell culture system (Huaxin Micro-fish, China) were used. The stage and image acquisition were controlled via NIS Elements software (Nikon, Japan), and the live-cell culture system had a one-touch start with automatic control of the culture conditions, including 37 °C, >98% humidity and 5% CO_2_. Bright-field and fluorescence images were acquired and analyzed via a customized MATLAB (MathWorks, USA) program. The algorithm extracts the position of nuclear centroids and the fluorescent intensity of nuclear and cytoplasmic regions, from which cell motility and fluorescent intensity were quantified.

### 2.7. Cell Viability Assessment and Statistical Analysis

Cell survival was assessed based on morphological integrity. Cells were counted as viable if they remained attached to the substrate, retained an intact outline, showed no fragmentation, and did not exhibit rounded, detached morphology characteristic of cell death. For each experimental condition, five culture chambers were selected per independent experiment, and each chamber was imaged at three predefined fields of view (proximal to the drug inlet, near the laminar interface, and distal from the interface). Imaging parameters were kept constant within each comparison group. Statistical significance was assessed using a *t*-test, with *p* < 0.05 considered significant.

## 3. Results

### 3.1. Design and Characterization of the Microfluidic Chip

To investigate how spatial heterogeneity within the cellular microenvironment influences cell behavior, we developed a microfluidic system capable of generating laminar flow within micron-scale culture chambers. The 64-chamber microfluidic system described here enables independent, dynamic control of the laminar interface position in each chamber through integrated pneumatic valves and on-chip peristaltic pumps. This setup allows the formation of two chemically distinct regions within a single cellular microenvironment, effectively mimicking the spatially heterogeneous chemical landscapes often encountered in vivo [30,31,32]. By leveraging the non-turbulent nature of low-Reynolds-number flow in microfluidic channels, this approach enables precise spatial control over chemical exposure at the scale of individual cells, offering a powerful tool for studying localized cellular responses to environmental cues.

To implement this concept, the microfluidic chip incorporates 64 identical culture chambers (Figure 1), each designed to support stable laminar flow at the microscale. The use of 64 chambers, arranged in an 8 × 8 array, facilitates high-throughput parallel experimentation, allowing simultaneous testing of multiple conditions with built-in replicates to enhance statistical robustness and minimize inter-chip variability. Each chamber is 25 μm in height and 400 μm in width, with inlet channels approximately 100 μm wide to promote uniform flow distribution and minimize hydrodynamic disturbances. It is independently addressable through row-column matrix control (Figure 1c), with integrated peristaltic pumps dynamically matching inlet flow rates to preserve interface stability across the array. While the data presented here focus on representative results for detailed analysis, this architecture supports large-scale parallel screening with improved condition-to-condition consistency compared to serial single-chamber approaches.

Each chamber is independently addressable through five integrated monolithic pneumatic valves (i.e., V-1 to V-5 as shown in Figure 1a), allowing flexible control over chemical input. Two separate inlets and one outlet are used to introduce distinct chemical streams, with valves 1 through 4 controlling the inputs. When valves 1–4 are open and valve 5 is closed, fluids from the two inlets simultaneously enter the culture chamber and establish a stable laminar interface, enabling localized chemical environments to be formed side-by-side within the same cellular microenvironment.

The relative position of the laminar-flow interface within each culture chamber is governed by the flow-rate ratio between the two inlets. To enable precise and tunable control of this interface, we integrated an array of microscale peristaltic pumps that regulate the input flow rates independently for each chamber (Figure 1b). These pumps achieve nanoliter-level delivery accuracy and are actuated through a sequence of digitally programmed pressure signals. Each peristaltic pump comprises three horizontal control lines and eight flow lines, operating in a cyclic sequence (e.g., 101 → 100 → 110 → 010 → 011 → 001), where a “1” indicates pressurization (valve open) and “0” indicates depressurization (valve closed). This actuation pattern generates directional flow through coordinated deformation of the flow channels. The resulting precise control over the volumetric flow from each inlet ensures that the laminar interface can be positioned accurately within the chamber, thereby defining sharp and controllable boundaries between two distinct chemical environments.

This real-time synchronization ensures that the two streams enter the culture chamber at matched velocities, preserving the stability of the laminar-flow interface regardless of the specific chemical composition delivered from input 1. As a result, distinct and reproducible chemical microenvironments can be established in each chamber, one at a time, with complete independence from neighboring chambers (Figure 1c). This modular and programmable structure allows for high-throughput screening of spatially resolved chemical cues under controlled conditions.

### 3.2. Continuous Introduction of Chemicals by Laminar Flows

**Figure 2:**
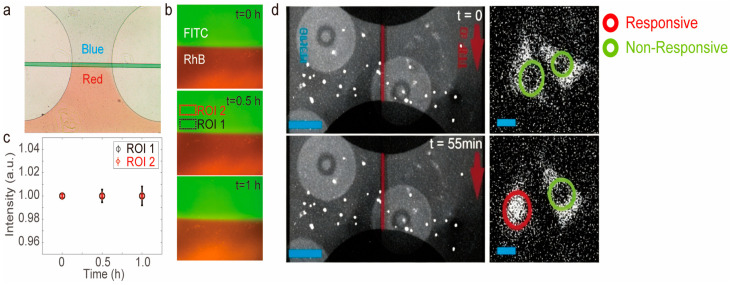
Laminar flows formed in a cell culture chip. (**a**) A bright-field image of the culture chamber shows that laminar flow can be formed at the center of the chip by controlling the input of two inlets. (**b**) The liquid containing fluorescent dye (i.e., fluorescein 5-isothiocyanate (FITC) and Rhodamine B (RhB)) forms a laminar flow in the culture chamber. It is demonstrated that the interface of the laminar flow can be well maintained for hours. (**c**) To verify the stabilization of the laminar flow in the culture chamber, two test windows were selected near the interface of the laminar flow, and the FITC fluorescence intensity of the windows was measured with an almost-constant intensity of ROI 2 (black, farther from the interface) and with slight fluctuations of ROI 1 (red, nearer the interface). (**d**) Fluorescent images show that cells located on the TNF-α-exposed side responded first, as expected. Cells on the opposite side, initially exposed only to culture medium, also exhibited NF-κB activation after a delay. Scale bar denotes 200 μm in (**d**), and 10 μm in the enlarged image.

To experimentally validate the formation and stability of laminar flow within the culture chambers, we introduced fluorescent dyes, i.e., Rhodamine B (red) and FITC (green), as well as food-grade colorants through the two inlets of each chamber. Upon actuation of the peristaltic pumps and appropriate valve configuration, the two streams entered simultaneously and formed a visible and sharply defined interface (Figure 2a), demonstrating the characteristic non-mixing behavior of laminar flow under low-Reynolds-number conditions.

To quantify the temporal stability of the laminar interface, we continuously monitored the spatial distribution of the fluorescent dyes over time (Figure 2b,c). Regions of interest (ROIs) were selected on either side of the interface: ROI 1 near the central boundary, and ROI 2 in a distal, well-separated zone. The fluorescence intensity of FITC in ROI 2 remained constant throughout the experiment, indicating highly stable chemical exposure in regions away from the interface. In contrast, ROI 1 exhibited only slight fluctuations, attributable to limited molecular diffusion at the interface. These results confirm that the device is capable of generating and maintaining stable laminar flow within each chamber over time, enabling robust control over localized chemical environments for cell stimulation (Figure 2d).

### 3.3. Spatiotemporal Profiling of TNF-α-Induced NF-κB Activation Mediated by Substrate-Level Diffusion

To experimentally validate the capability of our microfluidic chip in resolving spatially heterogeneous cellular responses, we cultured NIH 3T3 p65^−/−^ cells and stimulated them with tumor necrosis factor-alpha (TNF-α) under controlled laminar flow (Figure 2d and Supplementary Videos S1 and S2). TNF-α solution was introduced via one inlet, while standard culture medium was introduced via the other, establishing a stable chemical interface across the center of each chamber. At the flow rates employed, shear stress remained within the range previously shown to be innocuous to fibroblasts and keratinocytes; any loss of viability was therefore attributed to chemical exposure rather than mechanical stress. Fluorescence time-lapse imaging revealed a spatially dependent activation pattern: cells on the TNF-α-exposed side responded rapidly and with high synchrony (~50 min), cells near the laminar interface showed intermediate delay (~100 min), and cells at the distal end exhibited progressively staggered activation (>100 min) (Figure 2a and Figure S1). This differential response timing reflects the composite effects of molecular diffusion distance, local concentration buildup, and intracellular signal processing.

To investigate the mechanism behind this delayed response, we modeled TNF-α transport along the chamber bottom using one-dimensional lateral diffusion in a semi-infinite domain. The time-dependent concentration at position x and time t is described by the error function solution to Fick’s second law:

$$C\left(x,t\right)=C_{0}\cdot erfc\left(\frac{x}{2\sqrt{Dt}}\right)$$

where $C_{0}$ is the concentration at the TNF-α side (x=0), D is the diffusion coefficient of TNF-α in aqueous solution, and erfc denotes the complementary error function.

We estimated the diffusion coefficient D using the Stokes–Einstein relation:

$$D=\frac{k_{B}T}{6\pi \eta R}$$

with:

$k_{B}=1.38\times 10^{-23}$ J/K (Boltzmann constant),

T=310 K (37 °C, physiological temperature),

$\eta =0.69\times 10^{-3}\;Pa\backslash cdotps$ (viscosity of water),

R≈3.4 nm (hydrodynamic radius of TNF-α trimer, M≈51 kDa),

yielding:

$$D\approx \frac{1.38\times 10^{-23}\cdot 310}{6\pi \cdot 0.69\times 10^{-3}\cdot 3.4\times 10^{-9}}\approx 2\times 10^{-10}\;m^{2}/s$$

To estimate the time required for TNF-α to reach the far side of the 400 μm wide chamber (200 μm from the interface), we define two key time scales:

1. First-arrival time (digital triggering)—time for molecules to reach position x=200 μm:

$$t_{first}=\frac{x^{2}}{2D}=\frac{{\left(200\times 10^{-6}\right)}^{2}}{4\times 10^{-10}}=100\;s$$

2. Uniform threshold concentration time (analog triggering)—time for concentration at x=200 μm to reach 90% of $C_{0}$:

$$erfc\left(\frac{x}{2\sqrt{Dt}}\right)=0.9\Rightarrow \frac{x}{2\sqrt{Dt}}\approx 0.0886\Rightarrow t_{uniform}=\frac{x^{2}}{4D{\left(0.0886\right)}^{2}}\approx \frac{{\left(200\times 10^{-6}\right)}^{2}}{4.2\times 10^{-10}\cdot 0.00784996}\approx 6370\;s$$

These two regimes correspond to different cellular response modes. In a digital model, NF-κB is activated upon minimal TNF-α contact; the first-arrival time (~100 s) governs response. In contrast, in an analog model, activation depends on reaching a concentration threshold, governed by the uniformization time (~100 min). Our experimental results reflected both mechanisms: interface-adjacent cells responded rapidly, consistent with digital behavior; cells further away showed progressively delayed responses, consistent with analog activation. These findings are consistent with a proposed hybrid digital–analog model of NF-κB signaling. However, the relative contributions of ligand diffusion, receptor binding dynamics, and intracellular processing steps remain to be experimentally dissected.

Our results revealed a distinct spatiotemporal activation pattern of NF-κB in response to TNF-α under laminar-flow conditions. On the TNF-exposed side of the chamber, nearly all cells exhibited a synchronous nuclear translocation of NF-κB around 50 min after stimulation, consistent with a rapid, digital-like response. In contrast, cells on the opposite side, which were initially exposed only to culture medium, displayed delayed and progressively staggered activation. Starting approximately 100 min after stimulation, NF-κB nuclear translocation was first observed in cells located near the laminar interface and gradually extended toward the far end of the chamber.

Given that surface-level diffusion of TNF-α across the 200 μm distance is expected to reach a spatially uniform concentration within approximately 100 min, the significantly delayed cellular response observed on the medium side cannot be solely attributed to bulk diffusion along the substrate. One plausible explanation is that while TNF-α diffuses quickly along the chamber base, only a limited portion of the cell membrane is in close contact with the substrate, thereby constraining receptor–ligand interactions [33]. Consequently, TNF-α may need to further diffuse laterally across the cell membrane surface before sufficient receptor binding and signaling activation can occur, introducing additional delays.

While molecular diffusion is an intrinsic process governed by Fick’s law, the effective diffusive flux can be experimentally modulated by adjusting inlet concentration, flow rate, or interface position. These parameters alter the concentration gradient and diffusion path length, thereby tuning the spatial distribution of molecular exposure without changing the underlying diffusion coefficient. This distinction is important for interpreting the spatial response patterns observed in our assays.

This observation suggests that spatial features of cell–substrate interactions and membrane-localized cytokine diffusion play a critical role in determining the temporal dynamics of signaling, beyond classical bulk transport models [34,35]. Although our diffusion model captures the arrival of TNF-α at the cell surface, it does not incorporate the subsequent transcriptional and translational delays. These processes introduce additional latency distributions (typically tens of minutes for nascent p65-dsRed and >1 h for full reporter maturation) that overlay the spatial diffusion gradient. Consequently, the apparent ~100 min delay observed on the medium side is a composite of (i) ligand arrival, (ii) receptor binding, (iii) nuclear translocation, and (iv) reporter synthesis/maturation. Future kinetic models should explicitly include these steps to deconvolve diffusion from intracellular processing.

### 3.4. 5-FU Delivery Dynamics and Protective Effects in HaCaT–HSF Co-Cultures

**Figure 3:**
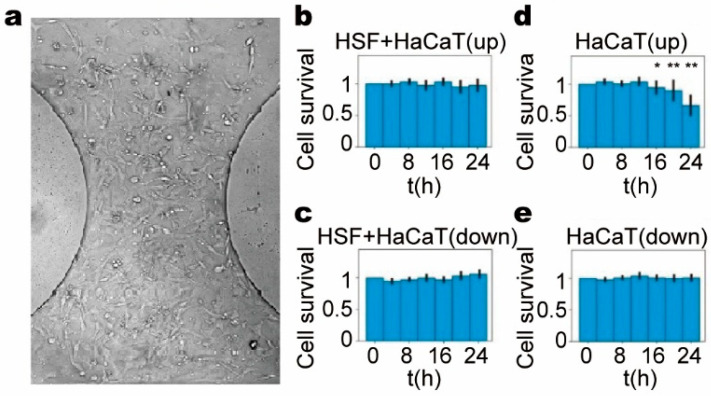
Evaluation of 5-FU delivery efficiency and fibroblast-mediated protection in a skin-mimetic microfluidic model. (**a**) Image of the culture chamber showing co-cultured HaCaT (keratinocytes) and HSF (human skin fibroblasts) cells. (**b**–**e**) Quantification of cell survival under different culture and flow conditions after 24 h exposure to 50 µM 5-FU under laminar flow: (**b**) Co-culture of HaCaT and HSF cells on the 5-FU inlet side shows limited cell death, indicating fibroblast-mediated protection. (**c**) Co-culture on the medium inlet side shows minimal cell death, consistent with low 5-FU diffusion. (**d**) HaCaT monoculture on the 5-FU side shows ~30% cell loss, demonstrating effective cytotoxicity without stromal protection. (**e**) HaCaT monoculture on the medium side exhibits negligible cell death, confirming insufficient diffusion to induce cytotoxicity. * *p* < 0.05, ** *p* < 0.01.

To further evaluate the chip’s capacity in mimicking in vivo skin structure, we established a HaCaT-HSF co-culture model exhibiting three-dimensional spatial organization and de novo TUBB3 expression, indicative of organoid-like properties (Figure 3 and Figure S2). To test the delivery efficiency of a model chemotherapeutic agent, we introduced 5-FU (50 µM) into one side of the culture chamber under laminar-flow conditions. In the monoculture of HaCaT cells, approximately 30% of cells in the drug-exposed region underwent cell death within 24 h (*p* < 0.01), while the distant end (culture medium inlet side) exhibited minimal cytotoxicity, confirming insufficient diffusion-driven drug accumulation to induce apoptosis at that location. In contrast, when HaCaT and HSF cells were co-cultured, cell death was significantly reduced even at the drug-inlet region, suggesting that fibroblasts conferred a protective effect on keratinocytes, potentially through paracrine secretion of survival factors, modification of the extracellular matrix, or direct cell–cell interactions. Prior studies have documented the protective roles of fibroblasts in epithelial chemoresistance and repair mechanisms in skin tissue models [36,37].

These results underscore the importance of including stromal components in organotypic skin models when evaluating drug delivery and efficacy. Moreover, they validate the chip as a platform not only for spatial drug diffusion studies but also for dissecting intercellular protective mechanisms in skin microenvironments. Existing microfluidic approaches typically achieve either gradient generation [24], droplet manipulation [22], laminar-flow dissolution studies [26], or cell capture [28], but rarely combine these capabilities. By integrating pneumatic control, 64-chamber independent addressability, and sub-chamber live-cell resolution, this platform enables dynamic stimulus routing with continuous optical access for spatiotemporal response mapping. It should be noted that the device is fabricated from PDMS, which can adsorb hydrophobic compounds; however, 5-FU is hydrophilic and TNF-α is a protein, both of which are expected to be minimally affected. For future studies involving hydrophobic drugs, low-sorption materials such as glass or surface-modified PDMS may be considered.

## 4. Conclusions

In this study, we developed and validated a laminar-flow microfluidic platform capable of precisely controlling spatial chemical microenvironments to investigate cellular responses. By exploiting low-Reynolds-number flow, we generated adjacent yet non-mixing fluid streams, thereby creating distinct chemical zones within the same culture chamber and enabling detailed dissection of spatiotemporal cellular behaviors.

The validation of laminar interface stability using fluorescent dyes (FITC and Rhodamine B) provides a foundation for interpreting the spatial distribution of bioactive molecules in our biological experiments. The diffusion coefficient of TNF-α is comparable to that of FITC, supporting the direct applicability of dye-based characterization to cytokine transport. For 5-FU, its diffusion coefficient exceeds that of the validation dyes, meaning that the limited penetration observed in our cytotoxicity assays represents a conservative estimate of drug distribution. These considerations strengthen the rigor of our biological conclusions.

Through NF-κB nuclear translocation assays in NIH 3T3 p65^−/−^ cells, we observed rapid and uniform responses in cells directly exposed to TNF-α, while delayed, gradient-like responses emerged on the medium side of the chamber. Mathematical modeling suggests that surface-level diffusion of TNF-α alone could account for the observed response onset within approximately 100 min. The apparent delay extending beyond this estimate may reflect additional constraints, such as limited ligand accessibility at the basal cell surface and subsequent lateral membrane diffusion, although these mechanisms remain speculative pending direct experimental validation.

Extending the platform to HaCaT–HSF co-culture, we show that 5-fluorouracil elicits spatially graded cytotoxicity in HaCaT keratinocytes, with cell death confined to regions proximal to the drug inlet when cultured alone. Inclusion of dermal fibroblasts markedly attenuates keratinocyte apoptosis even within the high-concentration zone. While this finding is consistent with paracrine or contact-dependent protective mechanisms reported in vivo, we acknowledge that additional controls (e.g., conditioned-medium experiments or indirect co-culture assays) would be required to establish the precise underlying mechanism.

Together, our results illustrate three central points: (i) laminar-flow microfluidics can resolve sub-chamber chemical gradients that faithfully mimic in vivo tissue heterogeneity; (ii) cellular signaling kinetics appear to involve an interplay between extrinsic diffusion and intrinsic membrane dynamics; and (iii) stromal–epithelial interactions appear to modulate drug sensitivity, suggesting that stromal components should be considered in predictive models of topical or systemic therapy.

Building on these capabilities, concrete pharmaceutical applications include spatial drug penetration profiling, stromal-mediated chemoresistance screening, and preclinical assessment of topical therapeutics in patient-derived tissue models. At the same time, these findings are subject to limitations, including simplified two-dimensional geometry, limited cell types, absence of immune and vascular components, and PDMS material properties such as hydrophobic compound adsorption. Additionally, while the 64-chamber design enables parallel experimentation with built-in replicates, systematic quantitative characterization of chamber-to-chamber reproducibility and long-term interface stability across the full array remains to be fully established and will be addressed in future validation studies. Although addressing these constraints would further enhance physiological relevance, this platform already offers a versatile framework for mechanistic studies of spatiotemporal signaling, high-content drug screening, and patient-specific evaluation of therapeutic efficacy under physiologically relevant microenvironmental conditions.

## Data Availability

The original contributions presented in this study are included in the article/Supplementary Material. Further inquiries can be directed to the corresponding authors.

## Supplementary Materials

The following supporting information can be downloaded at: https://www.mdpi.com/article/10.3390/mi17060655/s1, Videos S1 and S2: Time-lapse fluorescence imaging of TNF-α-induced NF-κB activation in NIH 3T3 p65^−/−^ cells under controlled laminar flow in a microfluidic chamber. Figure S1: Conceptual illustration of differential response timing across spatial positions. Figure S2: Spatial organization and phenotypic characterization of the HaCaT–HSF co-culture model.
