# Supplementary material for: A Laminar Microfluidic Platform for Probing the Effects of Spatially Heterogeneous Drug Distributions
Source: Micromachines (Basel). 2026 May 26;17(6):655. doi: 10.3390/mi17060655 (PMC13302910; doi:10.3390/mi17060655)
Supplement: Supplementary file 1 [file micromachines-17-00655-s001.zip › Supplementary Figures.pdf]

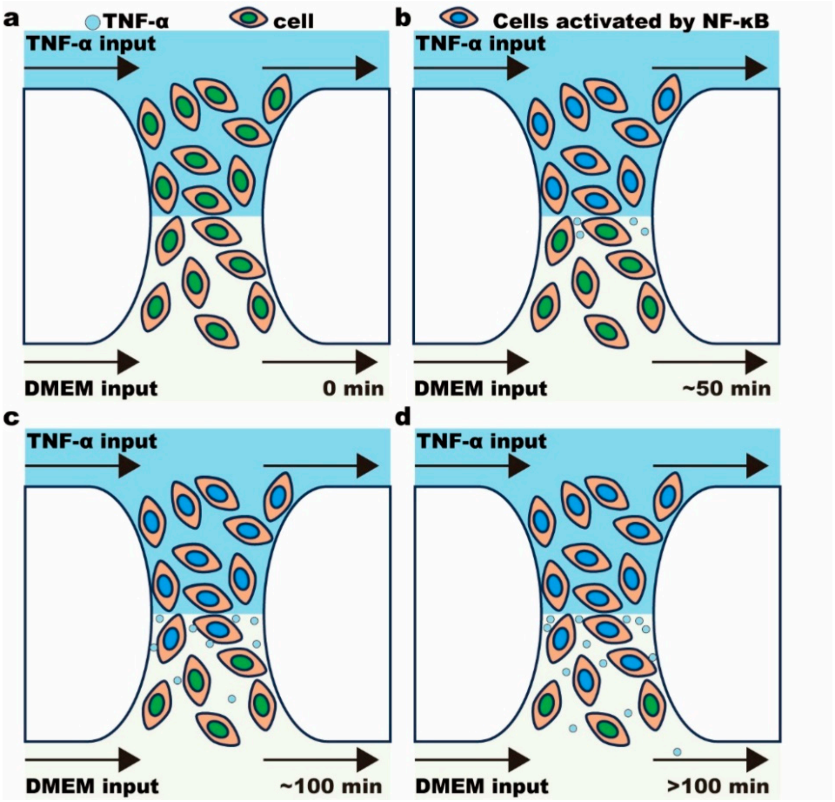

Figure S1. Conceptual illustration of differential response timing across spatial positions. (a) 0 min: stable laminar interface established. (b) ~50 min: rapid NF-κB activation in TNF-α-exposed cells. (c) ~100 min: delayed activation in interface-proximal cells. (d) >100 min: progressive activation extending toward distal regions. Time points are representative of experimental observations and reflect composite delays from molecular arrival, receptor engagement, and intracellular processing.

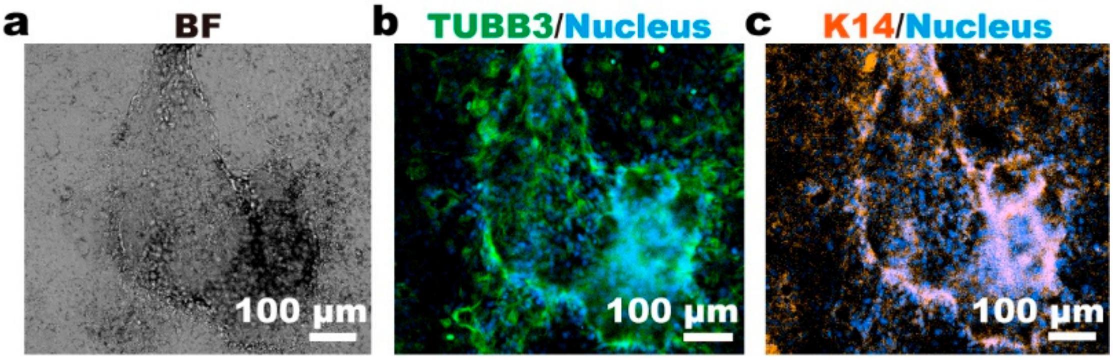

Figure S2. Spatial organization and phenotypic characterization of the HaCaT–HSF co-culture model. (a) Bright-field image showing three-dimensional aggregation and distinct spatial organization of the reconstructed skin-mimetic tissue, rather than simple two-dimensional monolayer spreading. (b) Immunofluorescence staining for tubulin beta-3 (TUBB3, Green). Neither HaCaT nor HSF cells express TUBB3 in monoculture; its de novo emergence following co-culture self-organization serves as a functional validation that the reconstructed tissue has acquired organoid-like properties. (c) Merged image showing the spatial relationship between K14-positive keratinocytes (Orange) and TUBB3 expression domains(Green). Scale bars: 100 μm.
